# Is Cooler Safer and More Advantageous? A Feasibility Study in Rabbits

**DOI:** 10.1093/ejcts/ezag012

**Published:** 2026-01-07

**Authors:** Emrah Şişli, Fatih Kar, Kıvanç İnan, Tarık Taştekin, Aykut Şahin, Dilek Çetinkaya, Dilek Burukoğlu Dönmez, Sema Uslu, Sadettin Dernek

**Affiliations:** Section of Paediatric Cardiovascular Surgery, Department of Cardiovascular Surgery, Osmangazi University Faculty of Medicine, Eskişehir, 26040, Turkey; Department of Biochemistry, Kütahya University of Health Sciences, Kütahya, Turkey; Eskişehir Sempati Animal Hospital, Eskişehir, 26130, Turkey; Department of Cardiovascular Surgery, Osmangazi University Faculty of Medicine, Eskişehir, 26040, Turkey; Department of Cardiovascular Surgery, Osmangazi University Faculty of Medicine, Eskişehir, 26040, Turkey; Department of Anaesthesiology and Reanimation, Osmangazi University Faculty of Medicine, Eskişehir, 26040, Turkey; Department of Histology and Embryology, Osmangazi University Faculty of Medicine, Eskişehir, 26040, Turkey; Department of Biochemistry, Osmangazi University Faculty of Medicine, Eskişehir, 26040, Turkey; Department of Cardiovascular Surgery, Osmangazi University Faculty of Medicine, Eskişehir, 26040, Turkey

**Keywords:** induced hypothermia, aortic coarctation, ischaemia, reperfusion injury, renal insufficiency, hepatic insufficiency

## Abstract

**Objectives:**

This study hypothesizes that induced hypothermia (IH) can safely extend aortic cross-clamp (ACC) time, significantly reducing metabolic burden and end-organ injury, thereby enabling more comprehensive aortic arch reconstructions and offering distinct advantages.

**Methods:**

In this experimental animal research, 62 New Zealand white rabbits were randomized into normothermia (39°C) and mild hypothermia (35°C) groups. Animals in each group underwent proximal aortic arch (PAA) clamping for 20, 30, or 40 minutes. Serial blood samples measured biochemistry, oxidative stress biomarkers, arterial and mixed venous blood gases, and lactate levels. Kidney and liver tissues were harvested for histopathological evaluation of ischaemic changes.

**Results:**

Normothermic rabbits experienced significant increases in oxidative stress, metabolic acidosis, and end-organ injury (renal, hepatic, and exclusively, spinal cord injury) with prolonged clamping. Conversely, IH markedly attenuated these adverse effects, preserving acid-base balance and reducing histological injury. Notably, the metabolic burden and end-organ injury observed after 30 minutes of hypothermic ischaemia were comparable with those after 20 minutes of normothermic ischaemia, suggesting a substantial extension of safe clamping time.

**Conclusions:**

Induced hypothermia during ACC provides significant protection against ischaemia-reperfusion injury by minimizing oxidative damage, preserving antioxidant capacity, and maintaining metabolic balance, thereby enhancing haemodynamic function post-surgery. This approach allows for safely extended ACC times, with a safety margin appearing to correspond to 30 minutes under hypothermia, facilitating complex aortic arch reconstructions and enabling safer surgical training. Clinical trials are warranted to confirm these findings in humans.

## Graphical abstract

**Figure:**
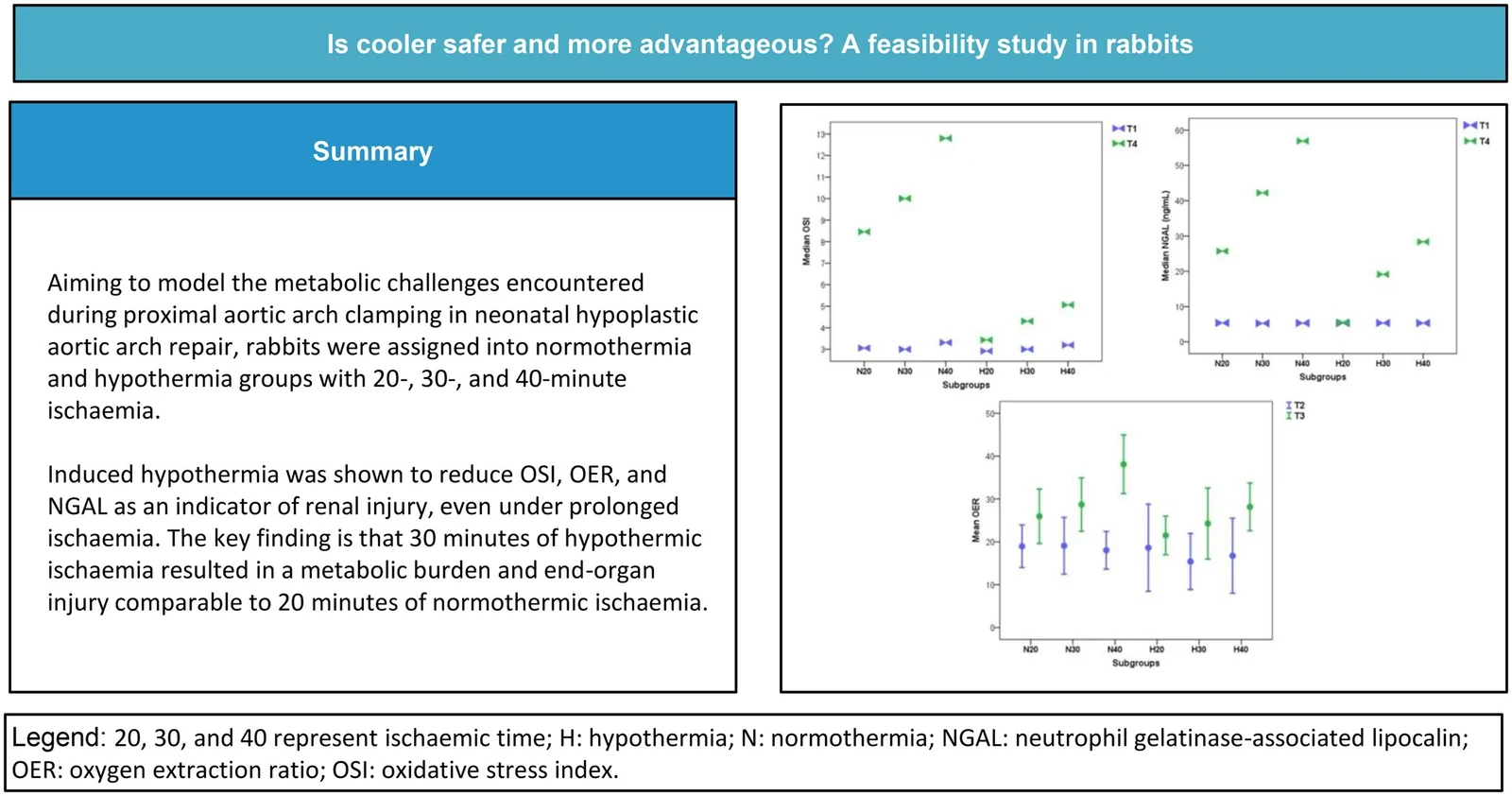

## Introduction

Neonatal coarctation of the aorta (CoA) frequently presents with aortic arch hypoplasia (AAH).^1–6^ While management of proximal aortic arch (PAA) involvement is variable,^3^^,^^5^^,^^7^^,^^8^ distal arch repair via left thoracotomy without cardiopulmonary bypass (CPB) requires aortic cross-clamping. Duration of aortic cross-clamp (ACC) typically ranges from 15 to 20 minutes but can extend to 40 minutes,^2^^,^^4^^,^^7^^,^^9–12^ leading to metabolic derangement and end-organ ischaemia.^4^^,^^7^^,^^9^^,^^11^ Prolonged aortic cross-clamping also impacts anastomosis quality^8^^,^^13^ and prevents trainees’ exposure.^14–16^

Mild hypothermia is defined as reduction of core temperature to 32°C-35°C.^17^ In humans, induced hypothermia (IH), typically 32°C-33°C, is employed in medicine to mitigate ischaemic injury by reducing metabolic demand.^17^ In clinical practice, while some utilize mild cooling (34-35°C), the application of deeper IH (32°C-33°C) for paediatric aortic arch surgery is not consistently reported.^2–12^^,^^18^^,^^19^ We hypothesize that IH can safely extend ACC time, enabling more extensive arch reconstructions, reducing metabolic burden, and mitigating end-organ injury, thereby offering significant advantages.

## Methods

### Ethical approval and animals

Ethical approval for this prospective study was obtained from Osmangazi University’s Local Ethics Committee (No: 783-1, March 2, 2022). All animal procedures adhered to the eighth edition of the *Guide for the Care and Use of Laboratory Animals*^20^ at Osmangazi University’s Animal Research Laboratory. Rabbits were housed individually in R-type cages at 22 ± 1°C, with a 12-hour light-dark cycle, and received a standard pellet diet and water. Sixty-two New Zealand white rabbits (no pre-existing cardiac lesions via echocardiography) were randomized into normothermia (N) or hypothermia (H) groups. Each group was further divided into 20-, 30-, and 40-minute ACC durations (N20, N30, N40, H20, H30, H40), with 10 rabbits per subgroup (H20 and H40 had 11). Aortic-cross-clamp durations were chosen based on evidence of increasing end-organ injury beyond 20 minutes.^4^^,^^7^^,^^9^^,^^11^

### Experimental protocol

**Figure 1.**
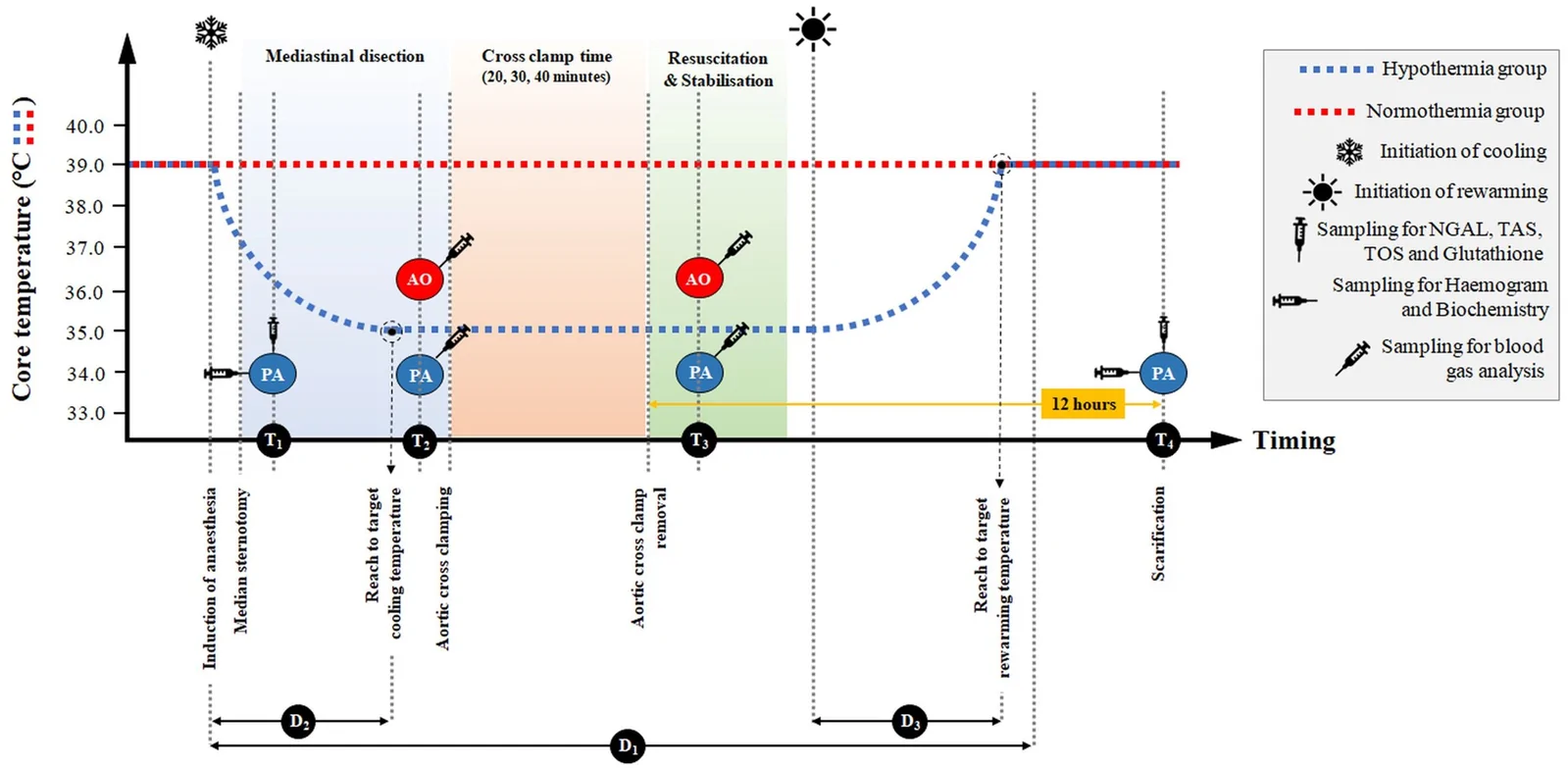
The Experimental Protocol Overview. Abbreviations: NGAL: neutrophil gelatinase-associated lipocalin; TAC: total antioxidant capacity; TOS: total oxidant status.

The experimental protocol is shown in **Figure 1**. Under physiological conditions, the body temperatures of the rabbits and humans are 39 °C and 36.5 °C, respectively.^17^^,^^20^ In clinical practice of IH in humans, core temperature is reduced by 10%-15%, which roughly corresponds to 35°C in rabbits and 33°C in humans.^17^ Accordingly, the protocol involved stabilizing the normothermia group at 39°C and cooling the hypothermia group to 35°C.

Following a 6-hour fasting, rabbits were weighed and received premedication (xylazine 1 mg/kg, ketamine 5 mg/kg, IV cefazolin 25 mg/kg). After cannulation and Carescape monitor B450 setup, anaesthesia (inspiratory fraction of 2% isoflurane, mechanical ventilation) was maintained, with tracheostomy performed (2 rabbits) if orotracheal intubation failed. Mild hypothermia was induced via an Arctic Sun 5000 system, with continuous rectal monitoring.

After median sternotomy, heparin (100 units/kg) was administered, the thymus was removed, and haemostasis was achieved. The pericardium was opened, and the ascending aorta and main pulmonary artery were prepared for blood sampling. After predetermined ischaemia through PAA clamping, ACC was gradually removed, with vital signs closely monitored. Stable rabbits underwent sternum closure, extubation, and were monitored postoperatively.

Twelve hours post-ACC removal (T_4_), rabbits underwent resternotomy for further blood and tissue sampling, followed by euthanasia via exsanguination. Before this second intervention and transfer to the operating room at T_4_, the neurological assessment of the rabbits was performed by a specialist veterinarian (BN), who was blinded to the research protocol. The neurological assessment included evaluating spinal cord injury (SCI) through the motor function of the hindlimbs, based on the International Standards for Neurological Classification of Spinal Cord Injury.^21^ Cases that showed full motor function were graded as 5 and categorized as “no SCI” whereas the cases that had partial or complete loss of motor function (grade 0-4) were categorized as “having SCI.”

### Biochemical measurements

Blood samples were taken at T_1_ (pre-ACC) and T_4_ (sacrifice) from the main pulmonary artery, with timing and sites shown in **Figure 1**. Full blood counts and biochemical tests (blood urea nitrogen [BUN]) and creatinine for renal function and aspartate transaminase (AST) and alanine transaminase (ALT) for hepatic function] were performed. Blood was analysed using automated systems. Serum was separated after centrifugation for biochemical parameters, stored at −80°C for neutrophil gelatinase-associated lipocalin (NGAL), total antioxidant capacity (TAC), total oxidant status (TOS), and glutathione analysis. The oxidative stress index (OSI) was calculated as TOS divided by TAC. Blood gas measurements were performed with a portable analyser (Radiometer ABL9). Arterial and venous oxygen content (CaO_2_ and CvO_2_) were calculated from arterial and mixed venous samples. Oxygen extraction ratios (OER) were calculated with the formula (SaO_2_ - SvO_2_)/SaO_2_ to assess oxygen consumption post-ACC.

### Histopathological evaluation

Kidney and liver tissues were fixed in 10% neutral-buffered formalin for 48 hours, then washed in tap water for 3-4 hours. After dehydration, they were cleared with two xylene baths of 20 minutes each. The samples were embedded in melted paraffin at 65°C for 60 minutes, sectioned at 5 micrometres, and mounted on slides. Following deparaffinization in xylene, they were stained with haematoxylin and eosin, dehydrated through graded alcohols, cleared in xylene, and mounted with entellan. Slides were examined by a blinded specialist (D.B.D.) under light microscopy (Olympus BH-2), and images were captured with an Olympus DP-70 camera. Seven images per subgroup were evaluated and scored for injury severity on a scale from 0 (no injury) to 3 (severe injury), as described previously.^22^ For renal injury, proximal, distal and collecting tubules, narrowing of Bowman’s space, and vascular congestion variables were scored. For liver injury, hydropic degeneration, necrosis, vascular congestion, oedema, thickening of the bile duct wall, and inflammation variables were scored.

### Statistical analysis

Power analysis was conducted during the study design to determine the sample size for each group based on potential mortality rates and was repeated post-hoc. The final analysis used a repeated measures design with 1 between-subject factor and 1 within-subject factor, involving 2 groups of 10 rabbits (totalling 20 subjects), with measurements at 3 time points. This design achieved 100% power to test the between-subject factor (factor B) using the Geisser-Greenhouse corrected F-test.

The Statistical Package for the Social Sciences (version 15.0, SPSS Inc., Chicago, IL, USA) was used for statistical analysis and graph generation. Normality was checked with histograms, Q-Q plots, and the Shapiro-Wilk test. Normally distributed variables are presented as mean ± standard deviation, with group comparisons using independent samples t-tests. Non-normal variables are presented as median (minimum-maximum), analysed with the Mann-Whitney *U*-test for between-group differences. Categorical data are presented as frequency (%) and compared with chi-square test or Fischer’s exact test. A *P* value ≤.05 was considered significant.

## Results

### Experiment and outcomes

The overall body weight of the rabbits was 2981 ± 119 gr and similar across all subgroups, with no significant differences. The study included 30 male (48.4%) and 32 female (51.6%) rabbits, with no significant differences observed between subgroups. The overall duration of the experiment (D_1_) was 69.2 ± 20.5 minutes (**Figures S1 and S2**). The difference in the initial body temperatures of the hypothermia and normothermia groups was not considerable (39.2 ± 0.3°C and 39.1 ± 0.2°C, respectively, *P* = .232). Specifically, within the hypothermia group, the time taken to reach the target temperature (D_2_) averaged 35.3 ± 3.1 minutes, while the mean duration of rewarming (D_3_) was 16.8 ± 2.4 minutes. Additionally, D_1_ in hypothermic subgroups was significantly longer than that of the normothermic subgroups with the same duration of ischaemia (*P* < .001 for each, **Figure S2**).

Two rabbits (1 from each of the H20 and H40 subgroups) died due to posterior wall rupture during PAA encircling after T_2_ prior to aortic cross-clamping. While their haemogram, biochemical, and oxidative stress data at T_1_, as well as blood gas analysis at T_2_ were included in the analysis, their corresponding results at T_3_ and T_4_ were missing. Among the other 10 mortalities resulting from post-clamp-release metabolic disturbances at T_3_, blood gas data at T_3_ were available; however, other laboratory and oxidative stress results at T_4_ were missing for analysis.

Excluding 2 technical deaths, overall mortality was 16.7% (10/60), with 26.7% (8/30) in normothermic versus 6.7% (2/30) in hypothermic groups (*P* = .080). No deaths occurred in N20, H20, and H30 subgroups. Subgroup mortality rates were 30% (3/10) in N30 versus 0% in H30 (*P* = .060), and 50% (5/10) in N40 versus 20% (2/10) in H40 (*P* = .350) (**Figure S3**). Critically, SCI occurred in 11 (22%) of surviving 50 rabbits, exclusively within normothermic subgroups (N30: 6/7, 85.7%; N40: 5/5, 100%). No hypothermic animals (*n* = 28) exhibited SCI. The rate of SCI was significantly higher in the normothermia group (50% [11/22] versus 0%, *P* < .001), with a considerable difference in subgroup analyses (*P* < .001 for overall normothermia versus hypothermia subgroups; *P* = .001 for N30 versus H30 and N40 versus H40).

### Biochemistry

**Figure 2.**
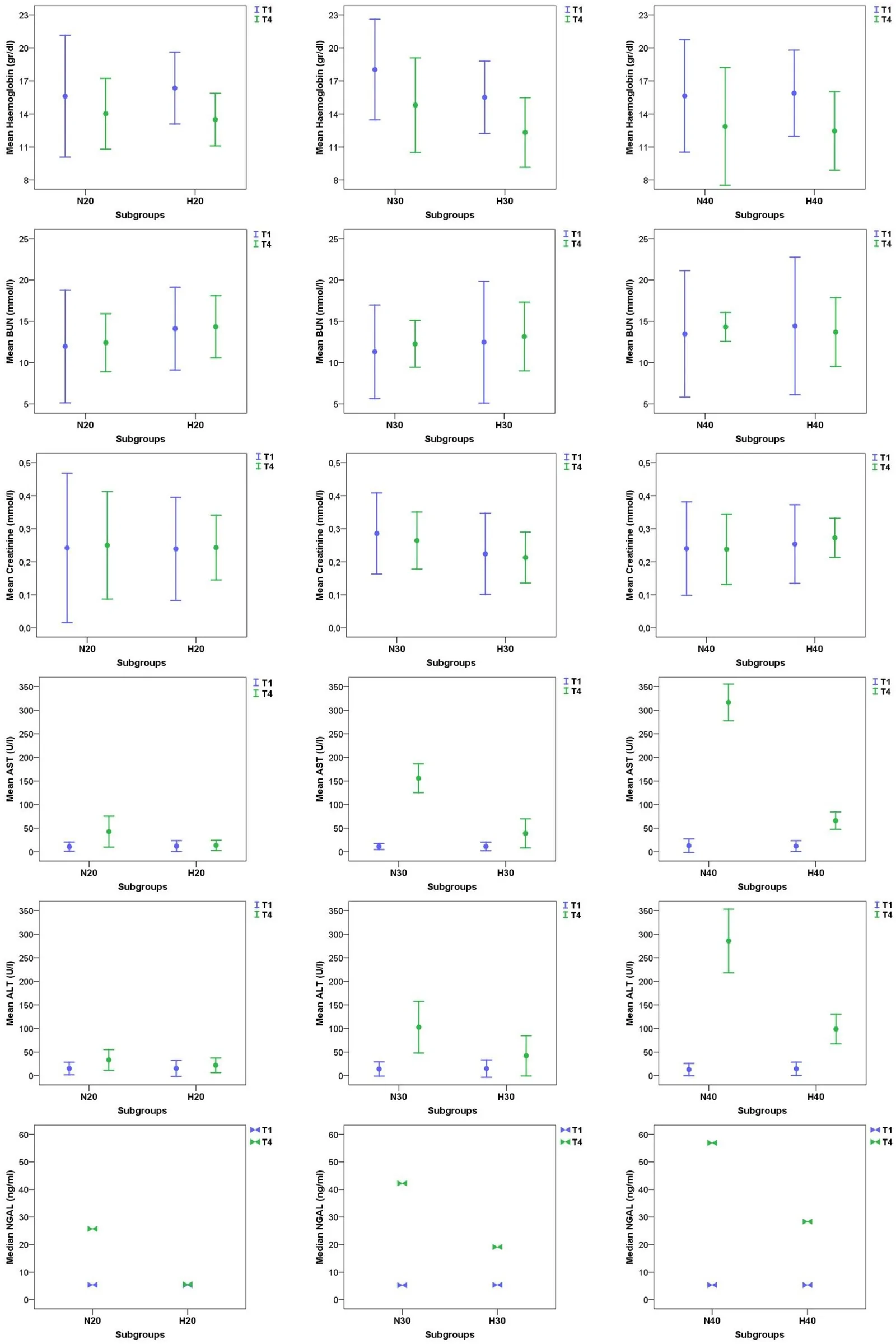
Laboratory Data (Haemogram, BUN, Creatinine, AST, ALT, and NGAL) at T_1_ and T_4_. Abbreviations: ALT: alanine transaminase; AST: aspartate transaminase; BUN: blood urea nitrogen; NGAL: neutrophil gelatinase-associated lipocalin.

The haemogram and biochemical values, along with comparative results, are detailed in **Table S1** and **Figure 2**. At both T_1_ and T_4_, haemoglobin did not show a significant difference between the corresponding same-duration of ischaemia subgroups, except N30, which was higher than H30 (*P* = .025). A substantial difference in BUN levels at T_4_ was observed between N20 and H20 (*P* = .028); however, there was no significant difference between other subgroup comparisons. Additionally, aside from the mean creatinine level at T_4_, which was higher in N30 compared to H30 (*P* = .027), there were no discernible differences among the subgroups. While NGAL levels showed no considerable difference between the subgroups at T_1_, all the NGAL levels at T_4_ were significantly higher in normothermic rabbits compared to hypothermic subgroups with same ischaemia time. Liver function tests (LFTs) revealed a progressive rise with the increasing duration of ischaemia in all subgroups. Besides, the mean AST and ALT levels were considerably higher in normothermia subgroups than in their corresponding hypothermia subgroups.

### Oxidative stress markers

**Figure 3.**
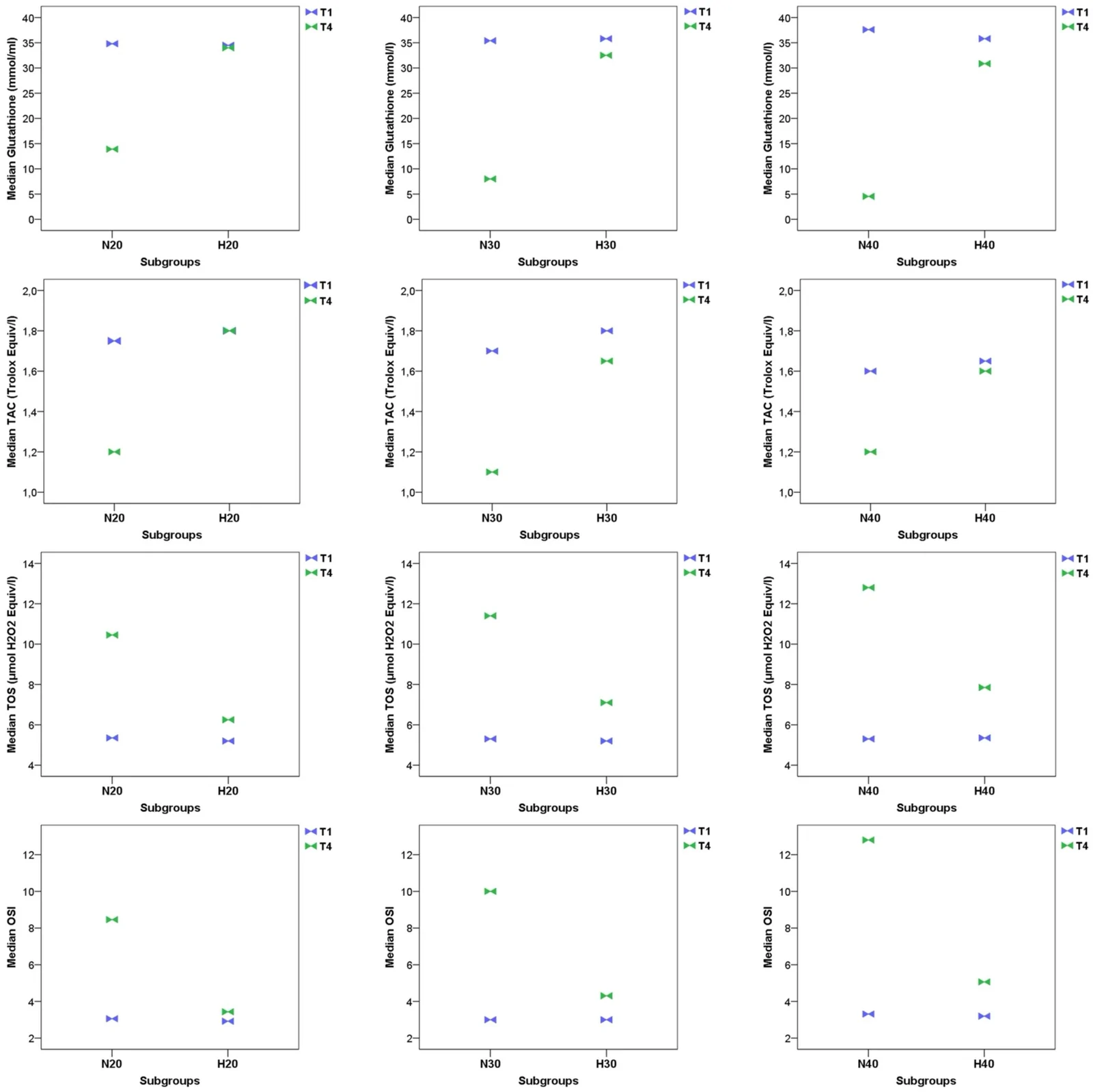
The Oxidative Stress Markers (Glutathione, TAC, TOS, and Oxidative Stress Index) at T_1_ and T_4_. Abbreviations: OSI: oxidative stress index; TAC: total antioxidant capacity; TOS: total oxidant status.

The results demonstrated significant changes in oxidative stress markers following the interventions (**Table S1** and **Figure 3**). Glutathione levels were around 35 nmol/mL at T_1_ in all subgroups without significant difference. However, they displayed an important differential pattern at T_4_: while the hypothermic subgroups maintained relatively stable levels at around 32 nmol/mL, normothermic groups showed significant reductions. In comparison to their counterparts, glutathione levels at T_4_ in hypothermic subgroups were significantly higher.

Total antioxidant capacity significantly decreased in normothermia subgroups, while there was no considerable decrease in hypothermic subgroups at T_4_. TAC was significantly higher in hypothermic subgroups relative to normothermic subgroups. Total oxidant status markedly elevated at T_4_ in all subgroups, but the rise was more pronounced in normothermic subgroups. In terms of OSI, there was no difference between the subgroups at T_1_ (*P* > .05). However, at T_4_, OSI was considerably higher among all normothermic subgroups.

### Blood gas analysis

**Figure 4.**
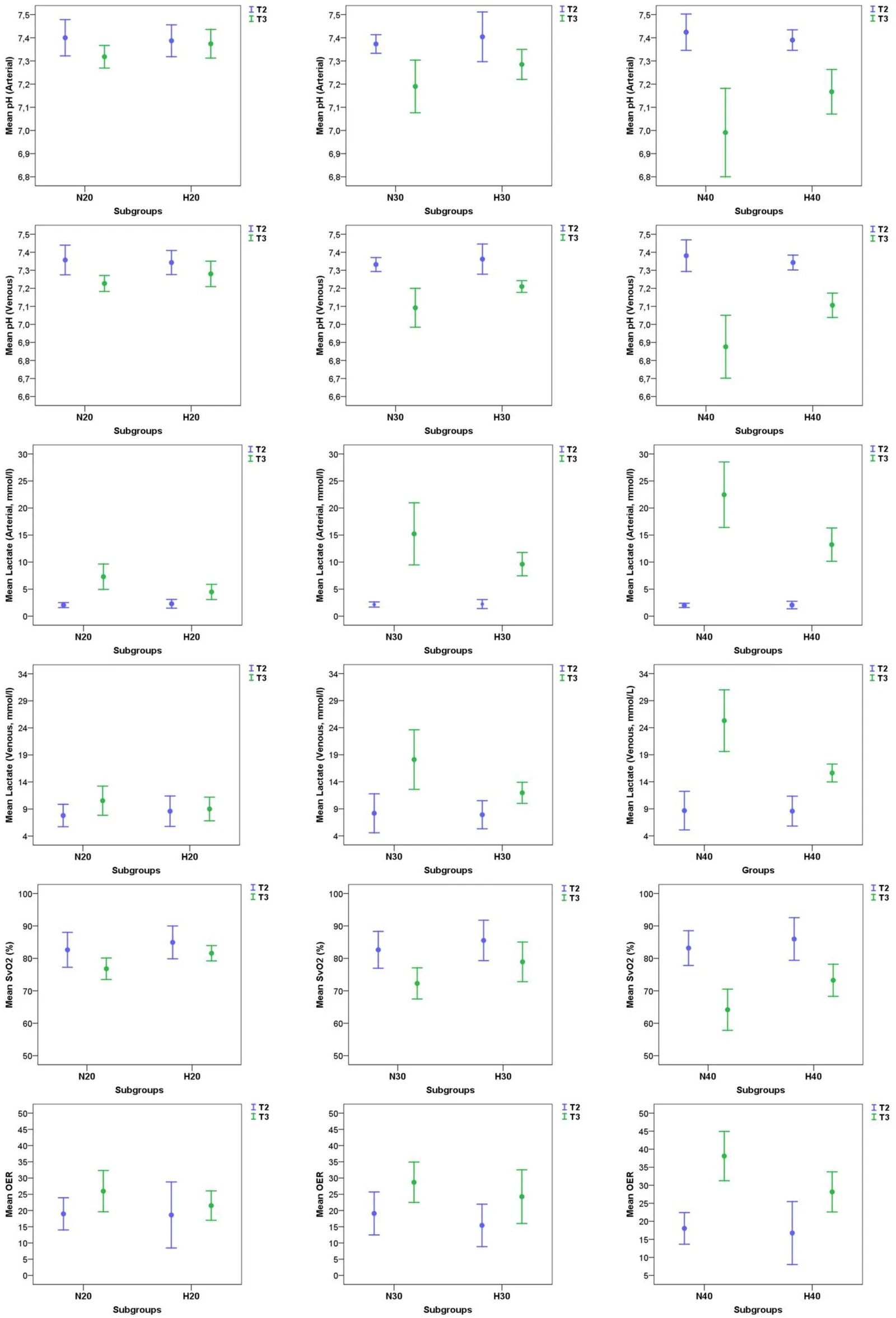
Arterial and Venous Blood Gas Analyses Results, Including Oxygen Extraction Ratio at T_2_ and T_3_. Abbreviation: OER: oxygen extraction ratio.

Significant differences in arterial and venous blood gases were observed between the normothermic and hypothermic subgroups at T_3_ (**Table S1, Figure 4**). The OER was comparable across subgroups at T_2_, except in between N30 and H30 (*P* = .022). At T_3_, the OER was significantly higher in normothermic subgroups, which was mainly due to the significantly higher SvO_2_ level in hypothermic subgroups where SaO_2_ did not differ. In terms of the lactate level and acidosis after clamp removal, while arterial and venous pH was significantly higher, both arterial and venous lactate levels were considerably lower in all hypothermic subgroups.

### Histopathology

**Figure 5.**
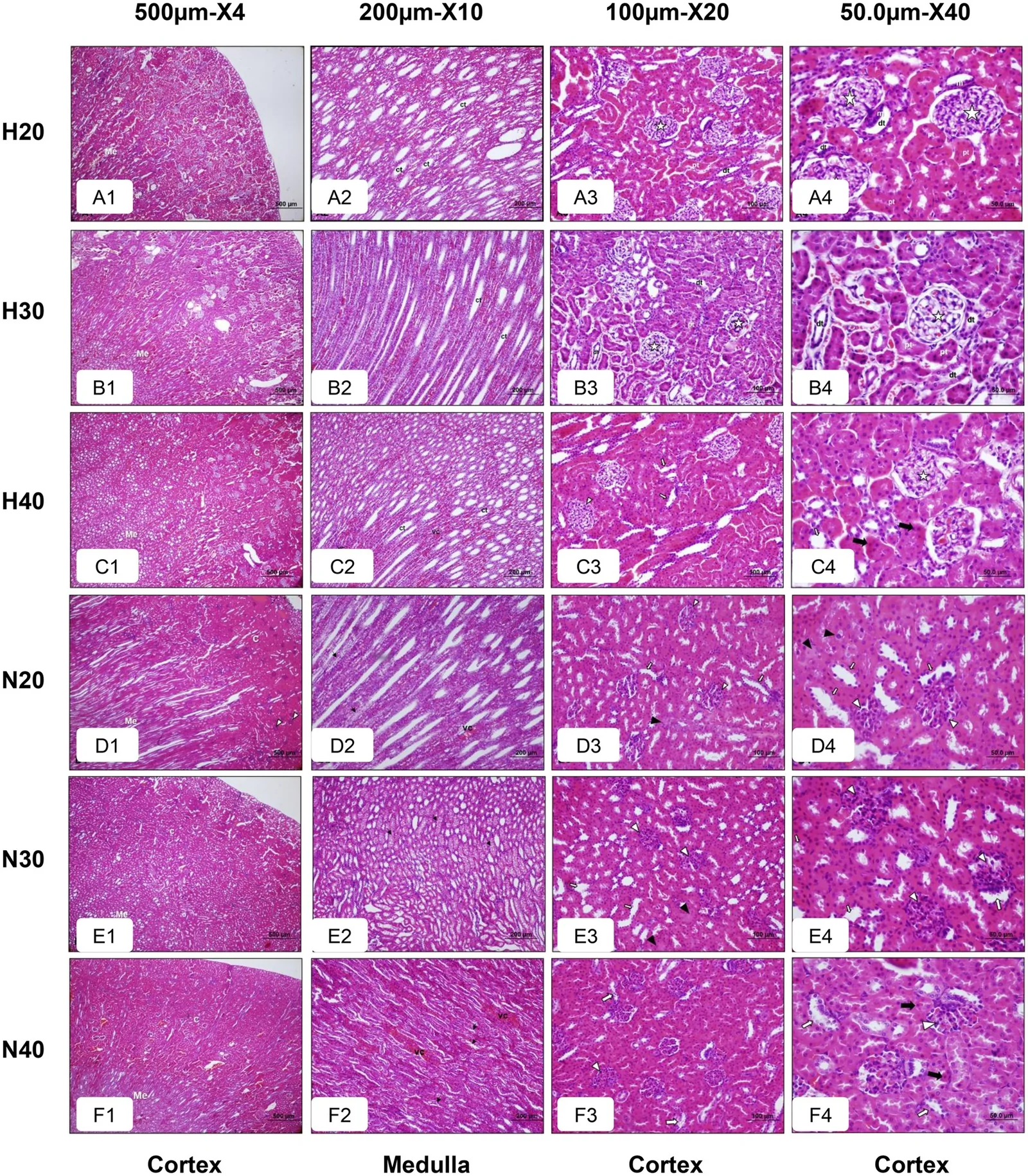
Haematoxylin-Eosin-Stained Light Microscopic Images of Rabbit Kidneys From All Experimental Groups at Different Magnifications. Indicators: (c) cortex, (Me) medulla, (
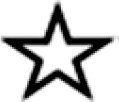
) Malpighian bodies, (dt) distal tubule, (pt) proximal tubule, (m) macula densa, and (ct) collecting tubules, vascular congestion (vc), narrowing of Bowman’s space (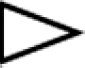), degeneration in distal tubule (
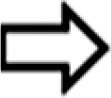
), necrotic cells with eosinophilic cytoplasm and pyknotic nuclei in the proximal tubules (
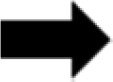
), vacuolar degeneration in collecting tubules (
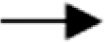
), necrotic cells in proximal tubules (
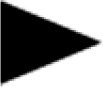
).

The histopathological assessment revealed significant differences in renal injury severity among the experimental subgroups (**Figure 5**, **Table S2**). Light microscopy images showed that the H20 and H30 subgroups largely preserved normal kidney morphology with intact cortex and medulla, well-preserved Malpighian bodies, proximal and distal tubules, macula densa, and collecting ducts. On the contrary, the H40 showed pathological changes, including narrowed Bowman spaces, degeneration of distal tubules, and necrotic tubules, and medullary vascular congestion with vacuolar degeneration, which were more pronounced in N40. In terms of injury scores, significantly higher scores were observed in the N40 and H40 subgroups compared to their related subgroups (*P* < .001 for total score). Specifically, proximal tubule injury scores increased from the median values of 1 (range 1-2) in N20 and H20 to 3 (range 2-3) in N40 and H40 subgroups, with similar trends observed in distal and collecting tubules. Bowman’s narrowing and vascular congestion also showed considerable rise in the N40 and H40 subgroups (*P* = .001), indicating more severe injury. Total injury score was markedly higher in the N40 subgroups compared to other subgroups.

**Figure 6.**
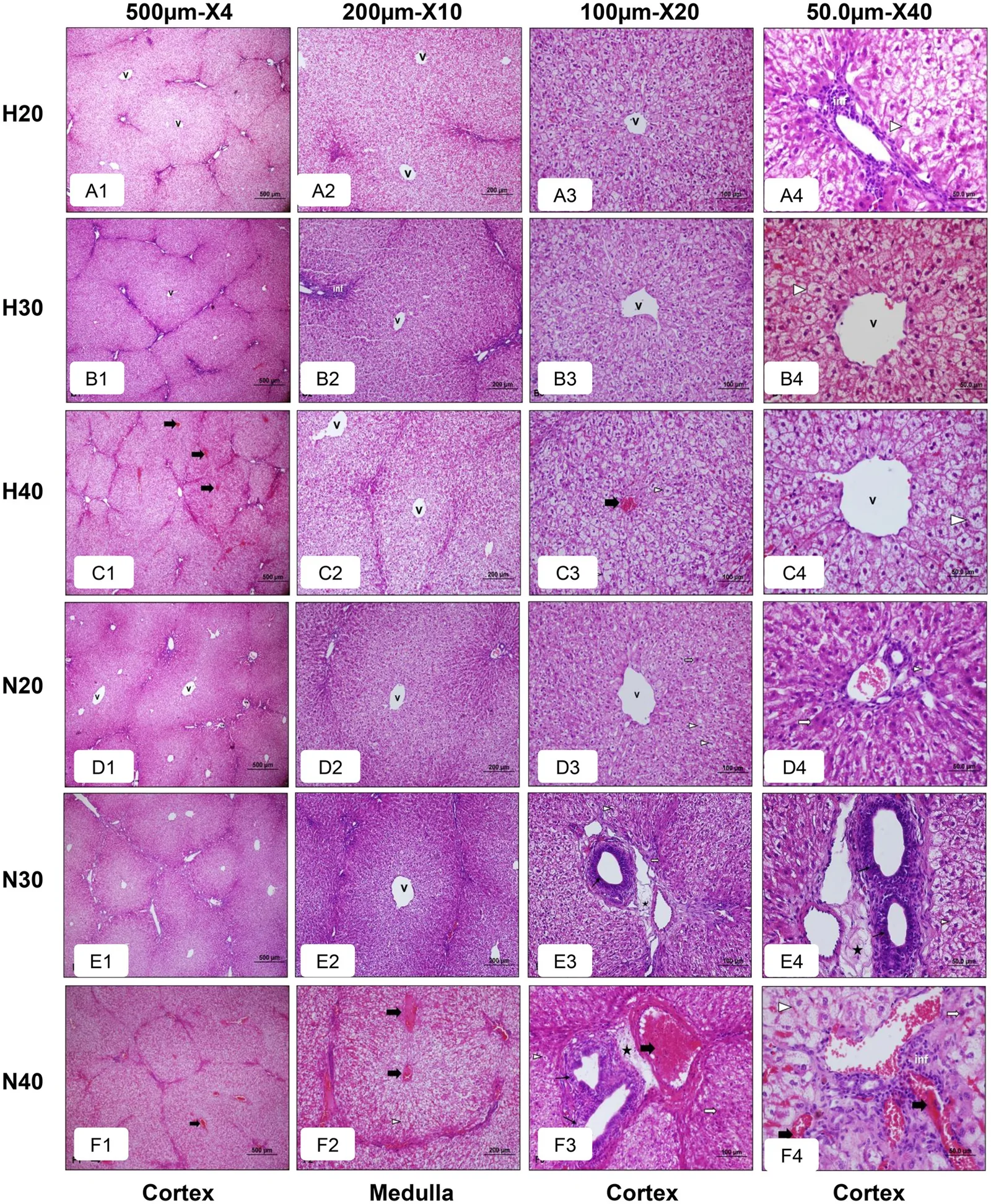
Light Microscopic Images of Rabbit Livers from All Experimental Groups at Different Magnifications. Indicators: inflammation (inf), vena centralis (v), hydropic degeneration (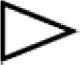), vascular congestion (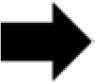), hepatocyte cells with eosinophilic cytoplasm and pyknotic nuclei (
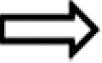
), oedema in portal region (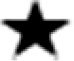), thickening of the bile duct wall (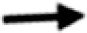).

The histopathological evaluation of rabbit livers (**Figure 6**, **Table S2**) demonstrated that the H20 and H30 retained near-normal liver architecture with minimal cellular inflammation and slight hydropic degeneration. Conversely, the H40 exhibited moderate hydropic degeneration, vascular congestion, and bile duct wall thickening, indicating increased injury. The N20 and N30 subgroups showed hepatocyte necrosis marked by pyknotic nuclei and eosinophilic cytoplasm, with N30 also displaying significant hydropic degeneration and bile duct thickening. The N40 presented with intense hydropic degeneration, pronounced vascular congestion, and notable hepatocyte changes. Quantitative injury scores corroborated these observations, with the N40 and H40 subgroups exhibiting significantly higher total scores compared to their corresponding subgroups.

### N20 versus H30 and N20 versus H40

These subgroup comparisons are detailed in **Tables S1 and S2** and **Figures 2**-**6**. Comparing N20 to H30, renal and hepatic enzymes were similar, but NGAL was significantly lower in H30 (*P* < .001). H30 also showed better oxidative stress markers (higher glutathione, TAC; lower TOS, OSI; all *P* < .001), despite N20 having slightly better arterial pH and lower lactate (*P* < .001). N20 exhibited higher renal injury scores histologically (*P* = .001). Conversely, N20 versus H40 revealed higher AST (*P* = .002), ALT (*P* < .001), and NGAL (*P* = .016) in H40, though oxidative stress markers favoured H40 (all *P* < .001). H40 had higher lactate and lower pH (*P* < .001) and increased total renal and liver injury scores histologically, including Bowman narrowing (*P* = .026) and oedema (*P* = .001), yet lower liver necrosis (*P* = .011).

## Discussion

Given the increased risk of end-organ damage with prolonged ortic cross-clamping,^4^^,^^7^^,^^9^^,^^11^ our key finding is that 30 minutes of hypothermic ischaemia resulted in a metabolic burden and end-organ injury (assessed biochemically, via oxidative stress markers, and histopathologically) comparable to 20 minutes of normothermic ischaemia (**Tables S1 and S2, Figures 5 and 6**). While H30 generally outperformed N20 across most parameters, H40 also maintained favourable oxidative stress profiles despite some metabolic challenges, affirming IH’s protective role.

As a therapeutic intervention in humans targeting to 32°C-33°C, IH is a recognized strategy to mitigate ischaemic injury by reducing metabolic demand in various clinical settings.^17^ However, specific recommendations for deeper IH (32°C-33°C) in paediatric aortic arch surgery remain limited, with many studies only reporting either surface cooling (34°C-35°C) via a blanket or omitting temperature management details.^2–12^^,^^18^^,^^19^ Contrary to surface cooling blankets, advanced temperature management systems provide tighter thermal control and real-time feedback, reducing safety risks linked to under- or over-cooling, such as electrolyte disturbance and arrhythmia.^23^

That said, we sought to demonstrate how IH could safely extend ACC time up to 40 minutes, as it would have many advantages: First, in addition to ischaemia/reperfusion (I/R) injury, it would provide less metabolic burden on patients. Second, it enables surgeons to perform more complex reconstructions in a less stressful environment without rush. Third, reduced concern over ischaemia duration would provide more opportunities for congenital cardiac surgery fellows to perform these index procedures, as it could safely permit a second aortic clamping in the same surgical session if anastomosis site bleeding or an unsatisfactory aortic opening occurs.

Cardiac surgery without CPB carries a significant acute kidney injury (AKI) risk between 7.4% and 38.4%.^7^^,^^24^^,^^25^ Our NGAL results (**Table S1**, **Figure 2**) and corresponding histopathology (**Table S2**, **Figure 5**) strongly indicate IH’s renal protective effect. Normothermic subgroups consistently showed higher NGAL and pronounced histological injury compared to hypothermic subgroups, where kidney morphology was largely preserved (H20, H30) or showed subtle changes (H40). Critically, NGAL levels in the N40 subgroup exceeded the significant kidney damage threshold for rabbits (>50 ng/mL^26^), while H40 did not, with a median level of 28.3 ng/mL. Consistent with the literature,^27^ this suggests IH effectively mitigates renal injury, allowing extension of aortic cross-clamping up to 40 minutes.

Similar protective effects were observed in the liver. AST and ALT levels, consistent with ischaemic liver injury severity,^28^ rose progressively with ischaemia duration in all subgroups but were significantly higher in normothermic subgroups. Hepatic enzyme trends and total injury scores (**Tables S1 and S2** and **Figure 6**) correlated with histopathological changes, confirming reduced tissue injury under hypothermic conditions and aligning with evidence that IH suppresses hepatic I/R injury.^28^

Our study notably found SCI exclusively in normothermic animals (85.7% in N30, 100% in N40), with no incidence in any hypothermic subgroup. This protection aligns with a previous abdominal aortic occlusion model,^29^ showing hindlimb motor dysfunction in 75% of 20- and 30-minute groups, and 87.5% in 40-minute group. The higher SCI rate in our N40 subgroup likely stems from our PAA clamping method, which causes more advanced spinal cord perfusion impairment.^30^ This highlights a crucial protective role for IH in preventing SCI during aortic arch clamping.

Ischaemia/reperfusion injury is driven by oxidative stress, characterized by energy depletion, hypoxanthine accumulation, simultaneous formation of xanthine oxidase, and subsequent reactive oxygen species generation upon reperfusion.^31^^,^^32^ Aortic cross-clamping induced significant oxidative stress, particularly in normothermic animals, manifesting as elevated TOS and OSI, and reduced TAC and glutathione (**Table S1** and **Figure 3**). These disturbances, most pronounced after 40 minutes of normothermic ischaemia, confirm hypothermia’s ability to reduce oxidative damage and preserve redox haemostasis,^31^ which are critical in neonatal aortic surgery. Furthermore, IH maintained superior acid-base balance and oxygenation (**Table S1** and **Figure 4**). While N40 animals developed profound metabolic acidosis with high lactate, all hypothermic subgroups maintained significantly higher pH and lower lactate levels, indicating more effective oxygen utilization and mitochondrial function, crucial given the limited metabolic reserve in neonates.^33^^,^^34^

Physiological assessment through arterial and mixed venous blood gas analysis further highlighted the protective role of IH in terms of acid-base haemostasis, the control of aerobic metabolism, and oxygenation balance in the experiment, reflecting the neonatal surgical setting (**Table S1** and **Figure 4**). Notably, in the N40 subgroup, normothermic animals developed profound metabolic acidosis with lactate levels during reperfusion. In contrast, compared with the normothermic subgroups, all hypothermic subgroups maintained significantly higher pH and lower lactate levels, indicating more effective oxygen utilization and better mitochondrial function. This is particularly critical in neonatal aortic arch repairs, where metabolic reserve is limited. Thus, these findings mirror the buffering benefits of IH in neonatal aortic procedures, where metabolic derangements can rapidly destabilize the neonate.

Induced hypothermia would empower surgeons to perform complex aortic arch reconstructions,^3^^,^^4^^,^^6^^,^^7^^,^^11^ such as patch aortoplasty, reverse subclavian flap, or roof enlargement under less time pressure, likely improving anastomosis quality and reducing reintervention rates. The urgency imposed by limited ACC time can lead to suboptimal outcomes, with reported recoarctation rates ranging from 8.6% to 20%.^5^^,^^8^^,^^12^ Kotani et al^8^ reported 8.6% early-stage recoarctation due to anastomotic stenosis. In Krylova et al’s^9^ study, re-application of ACC rate was 4.5% for bleeding, with an overall ACC time reaching 25 minutes. Reintervention rate before discharge in Rakhra’s cohort was 2.7%.^19^ Although some non-ischaemic clamping strategies extend procedure times to 40-43 minutes in roof enlargement techniques,^4^^,^^11^ ischaemic times were 23 minutes (18-32 minutes and 21-25 minutes), and declines in somatic near-infrared spectroscopy tracing indicate continued risk. Thus, particularly in complex cases with limited ductal flow, IH would offer significant advantages by extending safe working time for critical repairs. That said, as highlighted before, IH can safely permit a second aortic clamping in the same surgical session if anastomosis site bleeding or an unsatisfactory aortic opening occurs.

As for another advantage, the extended duration of ischaemia afforded by IH can improve surgical training for congenital cardiac fellows by allowing more hands-on experience in paediatric aortic arch surgeries, which are limited by time constraints and high risks.^14^^,^^15^^,^^35^ Supervising surgeons face a dilemma: optimize patient outcomes or provide training, often reducing fellows’ operative opportunities. Surveys reveal that 91% of graduates feel unprepared for independent practice due to limited mentorship, and only 36% are satisfied with their operative experience, with many operating in supportive roles.^14^ Furthermore, a structured 1- to 2-year transitional period post-fellowship is recommended to enhance readiness.^15^ Increasing safe ischaemia time would give trainees more opportunities to perform aortic arch procedures safely under supervision. In this context, IH offers a valuable step in the training phase, enabling safe learning before full independence.

That said, in terms of clinical relevance, the target population actually depends on the surgeon’s preference, and may comprise both simple and complex CoA cases with or without AAH. For instance, it can be used to protect end-organ injury and metabolic insult in more complex cases with longer ACC times, or even simple cases where reapplication of ACC is required due to anastomotic haemorrhage or inadequate luminal opening, which is around 4%-8% of the cases.^8^^,^^9^ Another target population would be patients in whom ACC time is predicted to be longer than 20 minutes, as a significant rate of CoA repairs takes more than 20 minutes.^4^^,^^9^^,^^11^ Additionally, it would be considered when a very inexperienced fellow performs coarctation repair for the first time. Overall, while IH has the potential to be applied to a wide range of patients on safety grounds, it is clearly not suitable when the proximal part of the PAA requires augmentation.

As an experimental animal research model, besides similarities of histopathological features, rabbits are phylogenetically closer to humans than rodents.^32^^,^^36^ In contrast, their vital signs and body temperature are quite different compared to humans.^32^^,^^37^^,^^38^ In terms of similarities peculiar to the current research, a rabbit’s spinal cord is prone to ischaemic injury much like the humans’.^29^ Additionally, as a source of reactive oxygen species in tissues exposed to I/R, xanthine oxidase is abundant in rabbit liver, much like it is in humans. NADPH oxidase and nitric oxide synthase are also readily available in rabbits, which results in the production of reactive oxygen radicals under I/R conditions.^31^^,^^32^ A body surface area (BSA) of an approximately 3 kg rabbit (the average body weight of our cohort) corresponds to 0.22 m^2^.^39^ Thus, it is clear that this represents the BSA of a newborn, which is the current study’s target population. Moreover, in addition to the cardiac output, which is around 200 mL/kg/minute in rabbits and 150-350 mL/kg/minute in newborns, the oxygen consumption of rabbits and humans is quite similar at around 6-8 mL/minute/kg.^37^^,^^38^^,^^40^ Despite their limitations, animal models for renal and hepatic I/R injury studies have been applied in rabbits for decades.^28^^,^^31^^,^^36^ Thus, due to the similarities in the metabolic response to I/R injury between rabbits and humans, rabbits were thought to be a suitable experimental model, albeit not exactly ideal.

Limited funding restricted further evaluations of urinary NGAL analysis and somatosensory/motor evoked potentials for SCI evidence. Directly measuring kidney and liver temperatures would have been valuable to demonstrate surface cooling effectiveness but was not performed due to anticipated morbidity. Measurements of metabolic rate could not be performed; however, calculating OSI, which reflects O_2_ bound to haemoglobin and dissolved in plasma, allowed an indirect assessment of oxygen consumption. Since the rabbits were sacrificed after 12 hours, the data are immediate-term, potentially limiting the study’s impact on the short- and intermediate term.

In conclusion, IH during ACC effectively protects against I/R injury, which is vital during neonatal surgeries. By reducing oxidative damage, preserving antioxidant capacity, and maintaining acid-base balance, IH mitigates risks of prolonged ACC times. This enables complex aortic reconstructions, provides a safer learning environment for trainees, and supports haemodynamic stability post-clamp release. Clinical trials are essential to translate these animal findings to human practice.

## Supplementary Material

ezag012_Supplementary_Data

## Data Availability

The data underlying this article will be shared on reasonable request to the corresponding author.
