# Erratum to: Is Cooler Safer and More Advantageous? A Feasibility Study in Rabbits

**DOI:** 10.1093/ejcts/ezag209

**Published:** 2026-08-20

This is an erratum to: Emrah Şişli, Fatih Kar, Kıvanç İnan, Tarık Taştekin, Aykut Şahin, Dilek Çetinkaya, Dilek Burukoğlu Dönmez, Sema Uslu, Sadettin Dernek, Is Cooler Safer and More Advantageous? A Feasibility Study in Rabbits, *European Journal of Cardio-Thoracic Surgery*, Volume 68, Issue 1, January 2026, ezag012, https://doi.org/10.1093/ejcts/ezag012

In the originally published version of this manuscript, Supplementary Figures 1, 2, and 3 were inadvertently omitted from the supplementary data file.

The figures have been added to the supplementary data zip file.

## Supplementary Material

ezag209_Supplementary_Data

