# Supplementary material for: Is Cooler Safer and More Advantageous? A Feasibility Study in Rabbits
Source: Eur J Cardiothorac Surg. 2026 Jan 7;68(1):ezag012. doi: 10.1093/ejcts/ezag012 (PMC12831934; doi:10.1093/ejcts/ezag012)
Supplement: ezag012_Supplementary_Data [file ezag012_supplementary_data.zip › Supplementary_Table_2.docx]

**Supplementary Table 2.** The histopathological injury scores of the kidney and liver within subgroups.

| **Variables** | **N20**  **(n= 10)** | **N30**  **(n= 7)** | **N40**  **(n= 5)** | **H20**  **(n= 10)** | **H30**  **(n= 10)** | **H40**  **(n= 8)** | **p value^b^**  **N20-H20** | **p value^b^**  **N30-H30** | **p value^b^**  **N40-H40** | **p value^b^**  **N20-H30** | **p value^b^**  **N20-H40** |
| --- | --- | --- | --- | --- | --- | --- | --- | --- | --- | --- | --- |
| ***Kidney Injury*** |  |  |  |  |  |  |  |  |  |  |  |
| Proximal tubule | 1 (1–2) | 2 (2–2) | 3 (2–3) | 0 (0–1) | 0 (0–1) | 2 (1–2) | **0.011** | **0.001** | **0.004** | **0.011** | 0.073 |
| Distal tubule | 2 (1–2) | 2 (2–3) | 3 (2–3) | 0 (0–0) | 0 (0–2) | 0 (0–2) | **0.001** | **0.004** | **0.017** | **0.038** | 0.902 |
| Collecting tubule | 1 (0–2) | 2 (1–2) | 3 (2–3) | 0 (0–0) | 0 (0–0) | 2 (0–2) | **0.004** | **0.001** | **0.007** | **0.004** | 0.259 |
| Bowman narrowing | 1 (1–2) | 2 (2–2) | 3 (3–3) | 0 (0–0) | 0 (0–1) | 2 (2–2) | **0.001** | **0.001** | **0.001** | **0.002** | **0.026** |
| Vascular congestion | 1 (1–2) | 2 (1–2) | 3 (2–3) | 0 (0–0) | 0 (0–0) | 1 (1–2) | **0.001** | **0.001** | **0.004** | **0.001** | 0.383 |
| Total score | 6 (5–9) | 10 (9–10) | 14 (12–15) | 0 (0–1) | 1 (0–3) | 9 (6–9) | **0.001** | **0.001** | **0.001** | **0.001** | **0.038** |
| ***Liver Injury*** |  |  |  |  |  |  |  |  |  |  |  |
| Hydropic degeneration | 1 (1–2) | 2 (2–2) | 3 (3–3) | 1 (0–1) | 1 (0–1) | 2 (1–2) | **0.128** | **0.001** | **0.001** | 0.259 | 0.073 |
| Necrosis | 1 (1–2) | 2 (2–3) | 2 (2–2) | 0 (0–0) | 0 (0–0) | 0 (0–1) | **0.001** | **0.001** | **0.001** | **0.001** | **0.011** |
| Vascular congestion | 0 (0–1) | 0 (0–1) | 3 (3–3) | 0 (0–0) | 0 (0–0) | 0 (0–0) | 0.383 | 0.383 | **0.001** | 0.383 | 0.383 |
| Oedema | 0 (0–1) | 1 (1–2) | 3 (2–3) | 0 (0–1) | 0 (0–1) | 2 (2–2) | 1.0 | **0.002** | **0.004** | 1.0 | **0.001** |
| Bile duct wall thickening | 0 (0–0) | 2 (1–2) | 3 (2–3) | 0 (0–0) | 0 (0–0) | 0 (0–1) | 1.0 | **0.001** | **0.001** | 1.0 | 0.383 |
| Inflammation | 0 (0–0) | 0 (0–1) | 3 (2–3) | 1 (0–1) | 1 (0–1) | 0 (0–1) | **0.026** | 0.073 | **0.001** | **0.004** | 0.209 |
| Total score | 3 (2–4) | 8 (7–9) | 16 (15–17) | 2 (1–2) | 2 (1–3) | 5 (4–6) | **0.017** | **0.001** | **0.001** | **0.053** | **0.004** |

^b^Mann Whitney U test. Note that score 0: no injury; 1: mild injury; 2: moderate injury; 3: severe injury.
