# Supplementary material for: Is Cooler Safer and More Advantageous? A Feasibility Study in Rabbits
Source: Eur J Cardiothorac Surg. 2026 Jan 7;68(1):ezag012. doi: 10.1093/ejcts/ezag012 (PMC12831934; doi:10.1093/ejcts/ezag012)
Supplement: ezag012_Supplementary_Data [file ezag012_supplementary_data.zip › Supplementary_Table_1.docx]

**Supplementary Table 1.** The biochemical, oxidative stress and blood gas analysis results in between the subgroups.

| **Variables** | **N20**  **(n=10)** | **N30**  **(n=10)** | **N40**  **(n=10)** | **H20**  **(n=11)** | **H30**  **(n=10)** | **H40**  **(n=11)** | **p value**  **N20-H20** | **p value**  **N30-H30** | **p value**  **N40-H40** | **p value**  **N20-H30** | **p value**  **N20-H40** |
| --- | --- | --- | --- | --- | --- | --- | --- | --- | --- | --- | --- |
| Haemoglobin (T_1_), *g/dl* | 15.6±2.8 | 16.6±3.1 | 15.9±2.8 | 16.5±1.6 | 15.5±1.6 | 15.8±1.7 | 0.395^a^ | 0.347^a^ | 0.529^a^ | 0.923^a^ | 0.888^a^ |
| Haemoglobin (T_4_), *g/dl* | 14.0±1.6 | 14.8±2.1**^α^** | 12.9±2.7^β^ | 13.5±1.2^λ^ | 12.3±1.6 | 12.5±1.8^Φ^ | 0.414^a^ | **0.025^a^** | 0.771^a^ | **0.028^a^** | 0.072^a^ |
| BUN (T_1_), *mmol/l* | 11.9±3.4 | 11.5±2.6 | 13.1±3.5 | 14.4±2.5 | 12.5±3.7 | 14.6±3.9 | 0.087^a^ | 0.479^a^ | 0.370^a^ | 0.752^a^ | 0.118^a^ |
| BUN (T_4_), *mmol/l* | 12.4±1.8 | 12.3±1.4**^α^** | 14.3±0.9^β^ | 14.4±1.9^λ^ | 13.2±2.1 | 13.7±2.1^Φ^ | **0.028^a^** | 0.310^a^ | 0.473^a^ | 0.395^a^ | 0.183^a^ |
| Creatinine (T_1_), *mmol/l* | 0.24±0.11 | 0.28±0.06 | 0.23±0.07 | 0.24±0.07 | 0.22±0.06 | 0.25±0.05 | 0.929^a^ | 0.087^a^ | 0.362^a^ | 0.665^a^ | 0.825^a^ |
| Creatinine (T_4_), *mmol/l* | 0.25±0.08 | 0.26±0.0**^α^** | 0.24±0.05^β^ | 0.24±0.05^λ^ | 0.21±0.04 | 0.27±0.03^Φ^ | 0.819^a^ | **0.027^a^** | 0.236^a^ | 0.216^a^ | 0.469^a^ |
| AST (T_1_), *U/l* | 10.9±4.8 | 12.2±4.4 | 12.4±6.4 | 12.0±5.5 | 11.4±4.5 | 12.4±6.3 | 0.617^a^ | 0.685^a^ | 0.987^a^ | 0.799^a^ | 0.539^a^ |
| AST (T_4_), *U/l* | 42.7±16.4 | 155.9±15.2**^α^** | 316.3±19.4^β^ | 13.6±5.4^λ^ | 39.1±15.4 | 66.1±9.2^Φ^ | **<0.001^a^** | **<0.001^a^** | **<0.001^a^** | 0.613^a^ | **0.002^a^** |
| ALT (T_1_), *U/l* | 15.3±6.7 | 14.2±6.6 | 13.7±5.5 | 14.7±8.5 | 15.1±9.2 | 14.2±7.0 | 0.870^a^ | 0.811^a^ | 0.848^a^ | 0.963^a^ | 0.724^a^ |
| ALT (T_4_), *U/l* | 33.4±10.9 | 102.7±27.4**^α^** | 285.5±33.7^β^ | 22.1±7.8^λ^ | 42.2±21.3 | 98.9±15.8^Φ^ | **0.017^a^** | **<0.001^a^** | **<0.001^a^** | 0.263^a^ | **<0.001**^a^ |
| NGAL (T_1_), *ng/ml* | 5.37  (4.35–6.04) | 5.11  (4.20–6.74) | 5.29  (4.95–6.14) | 5.16  (4.78–6.24) | 5.35  (3.97–6.45) | 5.20  (4.68–6.23) | 0.860^b^ | 0.910^b^ | 0.275^b^ | 1.0^b^ | 0.756^b^ |
| NGAL (T_4_), *ng/ml* | 25.69  (21.44–29.88) | 42.22  (40.64–43.28)**^α^** | 56.91  (55.87–59.18)^β^ | 5.56  (4.82–6.42)^λ^ | 19.12  (16.95–19.97) | 28.34  (26.41–29.64)^Φ^ | **<0.001^b^** | **0.001^b^** | **0.003^b^** | **<0.001^b^** | **0.016^b^** |
| TAC (T_1_), *mmol Trolox Equiv/l* | 1.8 (1.4–2.0) | 1.7 (1.4–2.0) | 1.6 (1.4–2.0) | 1.8 (1.6–2.0) | 1.8 (1.5–2.0) | 1.8 (1.5–2.0) | 0.431^b^ | 0.481^b^ | 0.352^b^ | 0.739^b^ | 0.918**^b^** |
| TAC (T_4_), *mmol Trolox Equiv/l* | 1.2 (1.2–1.3) | 1.1 (1.1–1.3)**^α^** | 1.2 (1.0–1.4)^β^ | 1.8 (1.5–1.9)^λ^ | 1.6 (1.5–1.6) | 1.6 (1.5–1.6)^Φ^ | **<0.001^b^** | **<0.001^b^** | **0.002^b^** | **<0.001^b^** | **<0.001^b^** |
| TOS (T_1_), *μmol H_2_O_2_ Equiv/l* | 5.4 (5.0–5.6) | 5.3 (5.0–5.7) | 5.2 (5.0–5.6) | 5.2 (5.0–5.6) | 5.2 (5.0–5.5) | 5.3 (5.0–5.6) | 0.413^b^ | 0.157^b^ | 0.497^b^ | 0.165^b^ | 0.973**^b^** |
| TOS (T_4_), *μmol H_2_O_2_ Equiv/l* | 10.5 (10.0–10.9) | 11.4 (11.0–11.8)**^α^** | 12.8 (12.4–14.0)^β^ | 6.3 (6.0–6.5)^λ^ | 7.1 (7.0–8.4) | 7.9 (7.4–8.4)^Φ^ | **<0.001^b^** | **<0.001^b^** | **0.003^b^** | **<0.001^b^** | **<0.001^b^** |
| Glutathione (T_1_), *nmol/ml* | 34.8 (31.5–36.2) | 35.5 (31.2–37.1) | 36.2 (30.8–38.1) | 34.8 (32.1–38.8) | 35.8 (31.9–38.7) | 35.5 (33.3–38.9) | 0.413^b^ | 0.157^b^ | 0.497^b^ | 0.218^b^ | 0.152^b^ |
| Glutathione (T_4_), *nmol/ml* | 13.9 (13-15) | 8 (7–9)**^α^** | 4.5 (2.0–5.6)^β^ | 34 (32–36)^λ^ | 32.5 (30.7–33.8) | 30.9 (28.1–32.6)^Φ^ | **<0.001^b^** | **0.001^b^** | **0.003^b^** | **<0.001^b^** | **<0.001^b^** |
| OSI (T_1_) | 3.1 (2.6–3.7) | 3.1 (2.6–4.0) | 3.2 (2.5–4.0) | 2.9 (2.5–3.2) | 3.0 (2.5–3.4) | 3.0 (2.6–3.7) | 0.244^b^ | 0.405^b^ | 0.549^b^ | 0.393^b^ | 0.973^b^ |
| OSI (T_4_) | 8.5 (8.1–9.0) | 10 (8.8–10.6)**^α^** | 12.8 (12.7–14)^β^ | 3.4 (3.2–4.0)^λ^ | 4.3 (3.9–4.5) | 5.1 (4.6–5.5)^Φ^ | **<0.001^b^** | **0.001^b^** | **0.003^b^** | **<0.001^b^** | **<0.001^b^** |
| ***Arterial blood gas*** |  |  |  |  |  |  |  |  |  |  |  |
| pH (T_2_) | 7.40±0.04 | 7.37±0.02 | 7.42±0.04 | 7.39±0.03 | 7.40±0.05 | 7.39±0.02 | 0.503^a^ | 0.104^a^ | **0.021^a^** | 0.851^a^ | 0.384^a^ |
| pH (T_3_) | 7.32±0.02 | 7.19±0.06 | 6.99±0.10 | 7.37±0.03^λ^ | 7.29±0.03 | 7.17±0.05^†^ | **<0.001^a^** | **<0.001^a^** | **<0.001^a^** | **0.020^a^** | **<0.001^a^** |
| Lactate (T_2_), *mmol/l* | 2.1±0.23 | 2.2±0.24 | 2.0±0.19 | 2.3±0.40 | 2.2±0.41 | 2.1±0.35 | 0.267^a^ | 0.602^a^ | 0.475^a^ | 0.222^a^ | 0.757^a^ |
| Lactate (T_3_), *mmol/l* | 7.3±1.17 | 15.2±2.87 | 22.5±3.03 | 4.5±0.70^λ^ | 9.6±1.08 | 13.2±1.54^†^ | **<0.001^a^** | **<0.001^a^** | **<0.001^a^** | **<0.001^a^** | **<0.001^a^** |
| SaO_2_ (T_2_), *%* | 99.5 (98–100) | 99.5 (97–100) | 98 (96–100) | 99 (96–99) | 98 (97–100) | 99 (97–100) | 0.352^b^ | 0.530^b^ | 0.157^b^ | **0.029^a^** | 0.251^b^ |
| SaO_2_ (T_3_), *%* | 98 (97–100) | 97.5 (96–100) | 98 (96–100) | 98 (96–99)^λ^ | 98 (94–100) | 98 (96–100)^†^ | 0.457^b^ | 0.695^b^ | 0.969^b^ | 0.529^a^ | 0.529^b^ |
| CaO_2_, (T_2_),ml O_2_/dl | 20.4±3.3 | 21.6±3.5 | 21.0±3.9 | 21.4±2.2 | 20.4±1.9 | 20.9±1.9 | 0.416^a^ | 0.375^a^ | 0.956^a^ | 0.991^a^ | 0.639^a^ |
| CaO_2_, (T_3_),ml O_2_/dl | 19.8±3.5 | 20.5±3.6 | 20.5±3.9 | 20.5±2.0^λ^ | 19.8±2.1 | 20.2±2.1^†^ | 0.617^a^ | 0.619^a^ | 0.835^a^ | 0.999^a^ | 0.782^a^ |
| ***Mixed venous blood gas*** |  |  |  |  |  |  |  |  |  |  |  |
| pH (T_2_) | 7.36±0.04 | 7.33±0.02 | 7.38±0.04 | 7.36±0.04 | 7.36±0.04 | 7.34±0.02 | 0.527^a^ | 0.054^a^ | **0.013^a^** | 0.791^a^ | 0.264^a^ |
| pH (T_3_) | 7.23±0.03 | 7.10±0.05 | 6.88±0.09 | 7.25±0.03^λ^ | 7.21±0.03 | 7.11±0.04^†^ | **0.001^a^** | **<0.001^a^** | **<0.001^a^** | 0.068^a^ | **<0.001^a^** |
| Lactate (T_2_), *mmol/l* | 7.8±1.0 | 8.2±1.8 | 8.7±1.8 | 8.4±1.5 | 7.9±1.3 | 8.6±1.3 | 0.275^a^ | 0.705^a^ | 0.879^a^ | 0.837^a^ | 0.153^a^ |
| Lactate (T_3_), *mmol/l* | 10.5±1.3 | 18.1±2.8 | 25.3±2.8 | 9.0±1.1^λ^ | 11.9±0.9 | 15.6±0.8^†^ | **0.013^a^** | **<0.001^a^** | **<0.001^a^** | **0.016^a^** | **<0.001^a^** |
| SvO_2_ (T_2_), *%* | 82.6±2.7 | 82.6±2.8 | 83.2±2.7 | 85.2±2.5 | 85.5±3.1 | 86.0±3.1 | **0.039^a^** | **0.044^a^** | **0.035^a^** | **0.039^a^** | **0.015^a^** |
| SvO_2_ (T_3_), *%* | 76.8±1.7 | 72.3±2.4 | 64.2±3.2 | 81.6±1.2^λ^ | 78.9±3.1 | 73.3±2.5^†^ | **<0.001^a^** | **<0.001^a^** | **<0.001^a^** | 0.070^a^ | **0.002^a^** |
| CvO_2_, (T_2_),ml O_2_/dl | 16.5±2.6 | 17.5±3.1 | 17.3±3.6 | 17.2±1.8 | 17.3±1.9 | 17.5±1.7 | 0.485^a^ | 0.856^a^ | 0.881^a^ | 0.465^a^ | 0.332^a^ |
| CvO_2_, (T_3_),ml O_2_/dl | 14.6±2.5 | 14.6±2.8 | 12.7±2.6 | 16.1±1.9^λ^ | 14.9±1.6 | 14.5±1.6^†^ | 0.162^a^ | 0.730^a^ | 0.084^a^ | 0.709^a^ | 0.882^a^ |
| OER (T_2_) | 18.9±2.5 | 19.1±3.3 | 18.0±2.2 | 18.6±5.1 | 15.4±3.3 | 16.8±4.4 | 0.851^a^ | **0.022^a^** | 0.420^a^ | **0.014^a^** | 0.185^a^ |
| OER (T_3_) | 25.9±3.2 | 28.7±3.1 | 38.1±3.4 | 21.5±2.3^λ^ | 24.3±4.1 | 28.2±2.8^†^ | **0.002^a^** | **0.015^a^** | **<0.001^a^** | 0.319^a^ | 0.118^a^ |

^a^Independent samples t test, ^b^Mann Whitney U test. Note that for the values at T_4_, there were seven rabbits in N30 (α), five in N40 (β), 10 in H20 (λ), eight in H40 (Φ), and 10 in H40 (†). **Abbreviations:** CaO_2_: arterial oxygen content, CvO_2_: venous oxygen content, SaO_2_: arterial oxygen saturation, SvO_2_: venous oxygen saturation, TAC: total anti-oxidant capacity, TOS: total oxidant status, OER: oxygen extraction ration, OSI: oxidative stress index.
