# Supplementary material for: Erratum to: Is Cooler Safer and More Advantageous? A Feasibility Study in Rabbits
Source: Eur J Cardiothorac Surg. 2026 Aug 20;68(8):ezag209. doi: 10.1093/ejcts/ezag209 (PMC13493045; doi:10.1093/ejcts/ezag209)
Supplement: Supplementary file 1 [file ezag209_supplementary_data.zip › Suppl Figure 2.pdf]

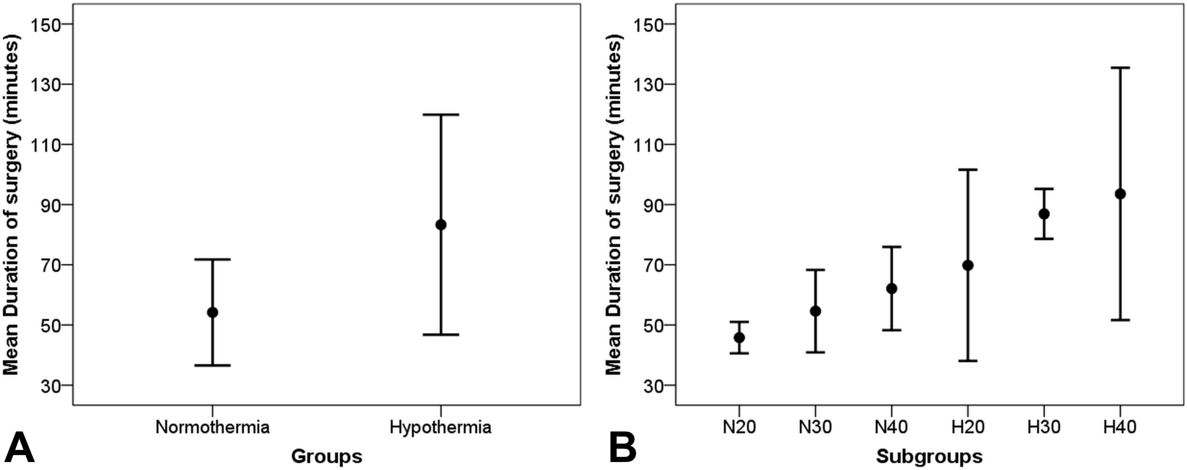

Mean Duration of surgery (minutes)
150
130
110
90
70
50
30
Normothermia
Hypothermia
Groups
A
Mean Duration of surgery (minutes)
150
130
110
90
70
50
30
N20
N30
N40
H20
H30
H40
Subgroups
B
