# Supplementary material for: Erratum to: Is Cooler Safer and More Advantageous? A Feasibility Study in Rabbits
Source: Eur J Cardiothorac Surg. 2026 Aug 20;68(8):ezag209. doi: 10.1093/ejcts/ezag209 (PMC13493045; doi:10.1093/ejcts/ezag209)
Supplement: Supplementary file 1 [file ezag209_supplementary_data.zip › Suppl. Figure 3.pdf]

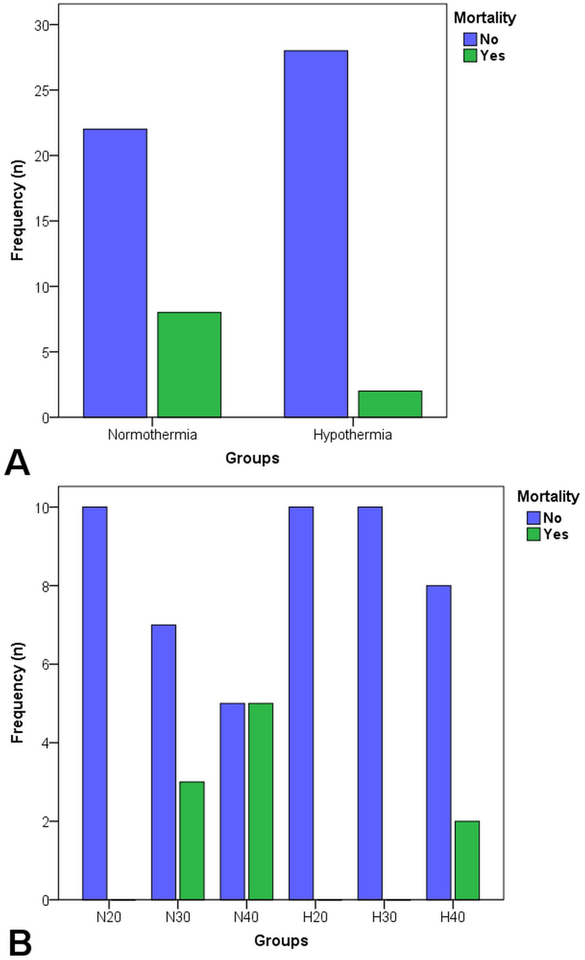

Mortality
No
Yes
Frequency (n)
0
5
10
15
20
25
30
Normothermia
Hypothermia
Groups
A
Mortality
No
Yes
Frequency (n)
0
2
4
6
8
10
N20
N30
N40
H20
H30
H40
Groups
B
